# Role of Sialyl-*O*-Acetyltransferase CASD1 on GD2 Ganglioside *O*-Acetylation in Breast Cancer Cells

**DOI:** 10.3390/cells10061468

**Published:** 2021-06-11

**Authors:** Sumeyye Cavdarli, Larissa Schröter, Malena Albers, Anna-Maria Baumann, Dorothée Vicogne, Jean-Marc Le Doussal, Martina Mühlenhoff, Philippe Delannoy, Sophie Groux-Degroote

**Affiliations:** 1 Univ Lille, CNRS, UMR 8576-UGSF- Unité de Glycosylation Structurale et Fonctionnelle, 59655 Villeneuve d’Ascq, France; sumeyye.cavdarli@univ-lille.fr (S.C.); dorothee.vicogne@univ-lille.fr (D.V.); philippe.delannoy@univ-lille.fr (P.D.); 2 Institute of Clinical Biochemistry, Hannover Medical School, 30623 Hannover, Germany; Schroeter.Larissa@mh-hannover.de (L.S.); Albers.Malena@mh-hannover.de (M.A.); anna-maria.junemann@gmx.de (A.-M.B.); Muehlenhoff.Martina@mh-hannover.de (M.M.); 3 OGD2 Pharma, IRS2—Nantes BIOTECH, 44200 Nantes, France; ledoussal@ogd2pharma.com

**Keywords:** ganglioside, sialic acid, *O*-acetylation, CASD1, *O*AcGD2, breast cancer

## Abstract

The *O*-acetylated form of GD2, almost exclusively expressed in cancerous tissues, is considered to be a promising therapeutic target for neuroectoderm-derived tumors, especially for breast cancer. Our recent data have shown that 9-*O*-acetylated GD2 (9-*O*AcGD2) is the major *O*-acetylated ganglioside species in breast cancer cells. In 2015, Baumann et al. proposed that Cas 1 domain containing 1 (CASD1), which is the only known human sialyl-*O*-acetyltransferase, plays a role in GD3 *O*-acetylation. However, the mechanisms of ganglioside *O*-acetylation remain poorly understood. The aim of this study was to determine the involvement of CASD1 in GD2 *O*-acetylation in breast cancer. The role of CASD1 in *O*AcGD2 synthesis was first demonstrated using wild type CHO and CHOΔ*Casd1* cells as cellular models. Overexpression using plasmid transfection and siRNA strategies was used to modulate CASD1 expression in SUM159PT breast cancer cell line. Our results showed that *O*AcGD2 expression was reduced in SUM159PT that was transiently depleted for CASD1 expression. Additionally, *O*AcGD2 expression was increased in SUM159PT cells transiently overexpressing CASD1. The modulation of CASD1 expression using transient transfection strategies provided interesting insights into the role of CASD1 in *O*AcGD2 and *O*AcGD3 biosynthesis, and it highlights the importance of further studies on *O*-acetylation mechanisms.

## 1. Introduction

Changes in cell surface glycosylation that affect both membrane glycolipids and glycoproteins occur during malignant transformation. Different cancer-associated glycans have been characterized so far as tumor associated carbohydrate antigens (TACA), and they are involved in the exacerbation of tumor aggressiveness [1]. In that light, complex gangliosides, such as GD2 and GD3, have been characterized as an oncofetal marker of melanoma [2] and neuroblastoma [3]. Besides, GD2 is also highly expressed in breast cancer (BC) patients with aggressive cancer subtypes [4]. Two glycosyltransferases, GD3 synthase (ST8Sia I, GD3S) and GD2 synthase (B4GALNT1, GD2S), control the biosynthesis of GD3 and GD2, respectively. Basically, gangliosides are acidic glycosphingolipids carrying one or more sialic acid residues in their carbohydrate moiety, and they are mainly located in lipid rafts at the outer leaflet of the plasma membrane [5]. They are found in different cell types as a mixture of di-, tri-, and tetra- saccharide structures, which confers to gangliosides a high structural heterogeneity [6]. Complex gangliosides from b- and c-series with two or more sialic acid residues linked to lactosyl-ceramide are usually absent from normal adult tissues, except the nervous system, but they are re-expressed in tumors from neuro-ectoderm origin where they exhibit a pro-tumoral action, mainly enhancing tumor aggressiveness through *cis*- and *trans*- interactions with tyrosine kinase receptors and the microenvironment [6,7,8]. We have previously shown that GD2 interacts with c-Met tyrosine kinase receptor in MDA-MB-231 BC cells and it induces the activation of PI3K/Akt and MEK/ERK signaling pathways [9]. When considering its expression and pro-tumoral activity in tumors from neuro-ectoderm origin, GD2 was extensively studied as target antigen for immunotherapy. In 2015, Dinutuximab (Unituxin^TM^) monoclonal antibody (mAb) has been approved by the Food Drug Administration for the treatment of pediatric high risk neuroblastoma [10]. However, the anti-GD2 mAb treatment caused severe side effects due to the expression of GD2 in healthy peripheral nerve fibers [11]. In parallel, the *O*-acetylated form of GD2 (*O*AcGD2) is exclusively expressed in cancer tissues [11], and it appears as an alternative target for cancer immunotherapy. It was shown that the anti-*O*AcGD2 c8B6 mAb induced in vitro mitochondrial cell death and cell cycle arrest in a mouse model of neuroblastoma, and decreased the tumor growth without inducing allodynia in vivo [12,13]. The biosynthesis of GD2 is very well described, but its mechanism of *O*-acetylation currently remains unclear.

**Figure 1:**
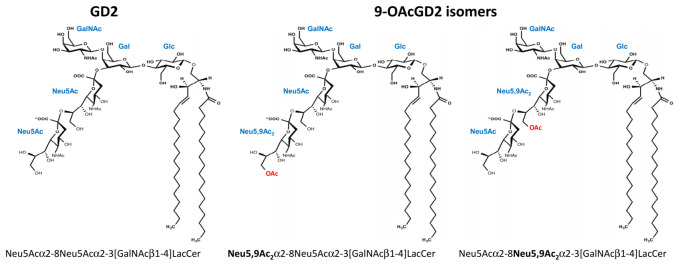
Structure of GD2 and *O*AcGD2 isomers identified in BC cells, with a 9-*O*-acetyl group (in red) either on the terminal or the subterminal sialic acid residue, resulting in Neu5,9Ac_2_α2-8Neu5Acα2-3[GalNAcβ1-4]LacCer or Neu5Acα2-8Neu5,9Ac_2_α2-3[GalNAcβ1-4]LacCer structures, respectively.

Ganglioside biosynthesis occurs in a stepwise manner by the sequential addition of glucose, galactose, N-acetylgalactosamine, and sialic acid residues on the ceramide moiety. GD2 is synthesized by the transfer of one N-acetylgalactosamine residue onto GD3. Our previous results suggest that GD2 can be converted into *O*AcGD2 by the addition of an acetyl group on a sialic acid residue by a sialate-*O*-acetyltransferase (SOAT); however, it is not clear which SOAT is involved in *O*AcGD2 synthesis. We previously analyzed the expression of *O*-acetylated and non-*O*-acetylated gangliosides in different cancer cell lines, and identified OAcGD2 expression in BC, melanoma, and neuroblastoma cells. MALDI-MS analysis showed that *O*-acetylation occurred either on the sub-terminal or the terminal sialic acid residue of the carbohydrate moiety [14]. Sialic acids are a family of 9-carbon monosaccharides that are derived from neuraminic acid (Neu5Ac) that can be acetylated the OH group of carbon -4, -7, -8, or -9 [15,16]. We have determined the precise position of *O*-acetyl substitution on sialic acid residue in BC gangliosides and shown that gangliosides that are expressed by BC cells are mainly acetylated on the carbon 9, forming Neu5,9Ac_2_, which suggests that BC cells mainly express 9-*O*AcGD2 [17]. Two *O*AcGD2 isomers were identified by MS/MS fragmentation in BC cells, with the *O*-acetyl group either on the terminal or internal sialic acid residue (Figure 1).

*O*-acetylation of gangliosides takes place in the Golgi apparatus in a cell- and development-dependent manner [18]. Different levels of regulation, including substrates availability, Golgi-ER transporter, and the balance between sialyl-*O*-acetyltransferase (SOAT) and sialyl-*O*-acetylesterase (SIAE) activities, control this process [19,20]. All of the attempts made for the biochemical isolation of mammalian SOATs were unsuccessful and, over decades, the genetic basis of mammalian SOATs remained elusive [21,22]. In 2011, Arming and coworkers performed a database-mining approach, which finally led to the identification of *C*ASD1 (Cas1 domain containing 1) as a putative human SOAT [23]. CASD1 shares sequence similarity with Cas1 (capsule synthesis 1) of the fungal pathogen *Cryptococcus neoformans*, which catalyzes the transfer of *O*-acetyl groups at the C6 position of mannose residues of the cryptococcal capsular polysaccharide glucuronoxylomannan [24].

**Figure 2:**
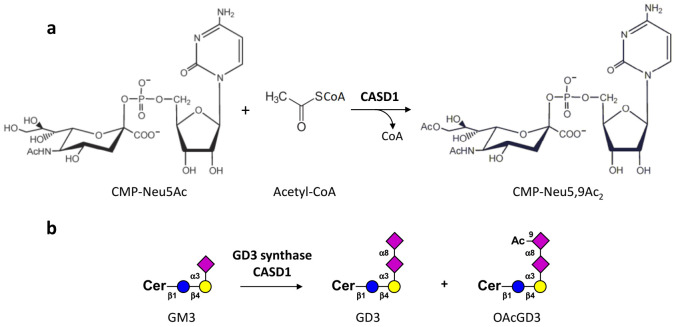
Current knowledge on CASD1 sialate-*O*-acetyltransferase activity. (**a**) CASD1 SOAT activity on CMP sialic acid, generating CMP-Neu5,9Ac_2_ was proposed by Baumann et al., 2015 [25] (**b**) In HAP-1 cells, the expression of GD3S induced the formation of GD3 and 9-*O*AcGD3.

The human *CASD1* gene consists of 18 exons on chromosome 7q21.3. The major transcript encompasses 3942 nucleotides and encodes a 797 amino-acid protein that is composed of an N-terminal serine-glycine-asparagine-histidine (SGNH) hydrolase-fold domain that harbors a catalytic triad and a C-terminal multipass transmembrane domain [25]. CASD1 is localized in the Golgi apparatus with its SGNH domain facing the Golgi lumen, as shown by subcellular localization and selective membrane permeabilization [25]. In vitro studies with the purified SGNH domain of CASD1 demonstrated the transfer of acetyl groups from acetyl-coenzyme A to position C9 of CMP-activated sialic acid (Figure 2a), which provided direct evidence for SOAT activity. The selective gene knockout in HAP-1 and HEK293 cells confirmed a critical role of CASD1 in sialic acid 9-*O*-acetylation [25]. In line with this, the initial analysis of *Casd1^−/−^* mice showed a complete loss of (7), 9-*O*-acetylation of sialic acid on the surface of hematopoietic lineage cells, such as myeloid, erythroid, and CD4+ T cells [26]. Potential changes in the ganglioside pattern of *Casd1^−/−^* mice have not yet been addressed and, so far, the role of CASD1 in ganglioside *O*-acetylation has been only studied in the context of GD3. The overexpression of CASD1 and GD3S in COS cells correlated with an increase in 7-*O*AcGD3 biosynthesis [23]. In HAP-1 cells, the expression of GD3S induced the formation of GD3 and 9-*O*AcGD3 (Figure 2b). The latter was lost in GD3S-expressing HAP-1Δ*CASD1* cells, demonstrating a crucial role of CASD1 in the biosynthesis of 9-*O*AcGD3 [25]. Whether CASD1 is also required for the *O*-acetylation of more complex gangliosides, such as GD2, has not been explored.

In this study, we aimed at defining the role of CASD1 in GD2 *O*-acetylation in engineered CHO and SUM159PT BC cell lines. *CASD1* expression was modulated in SUM159PT cells using plasmid transfection for overexpression and siRNA and shRNA strategies for gene silencing. We show that *O*AcGD2 expression was reduced in SUM159PT that was transiently depleted for CASD1 expression. In parallel, *O*AcGD2 expression was increased in SUM159PT cells transiently overexpressing CASD1. The role of CASD1 in *O*AcGD2 synthesis was dissected in CHO cells. The co-expression of GD3S and GD2S induced the formation of 9-*O*-acetylated GD2 in CHO wild type, but not in CHOΔ*Casd1* cells. These data show that CASD1 is essential for the biosynthesis of 9-*O*AcGD2.

## 2. Materials and Methods

### 2.1. Antibodies

The anti-GD3 R24 mouse IgG3 was purchased from Abcam (Cambridge, MA, USA). The mouse IgM anti-9-*O*AcGD3 mAb M-T6004 was from Thermo Scientific (Waltham, MA, USA). The anti-GD2 mAb 14.18 mouse IgG3/k and anti-*O*AcGD2 mAb 8B6 mouse IgG3/k were produced in CHO cells by OGD2 Pharma (Nantes, France). The mouse IgG2a anti-GD2 mAb ME361 that was used for immune-TLC experiments was from Kerafast (Winston-Salem, NC, USA). The secondary antibodies Alexa Fluor 488 donkey anti-mouse IgG and Alexa Fluor 546 donkey anti-rabbit IgG were purchased from Invitrogen (Cergy Pontoise, France).

### 2.2. Mammalian Expression Plasmids

The plasmids pcDNA3.1-zeo-V5-ST8SIA1 and pcDNA3-V5-CASD1-Myc were generated as described previously [25]. For efficient co-expression of GD3S and GD2S, we generated a plasmid that carries the coding sequence of GD3S (accession no. NM_011374.2) without stop-codon fused to a sequence stretch that encodes the self-cleaving 2A peptide of equine rhinitis A virus (QCTNYALLKLAGDVESNPGP) and the coding sequence of GD2S (accession no. NM_008080.5). The entire tripartite sequence was generated by gene synthesis (Eurofins MWG Operon), amplified by PCR using the primers 5′-ATAGCGGCCGCATGAGCCCCTGCGG-3′ and 5′-GCTCTCTAGATCACTCGGCGGTCATGCAC-3′, and the obtained PCR product was ligated into the NotI and XbaI restriction sites of the vector pcDNA3 (Invitrogen). The identity of the final construct was verified by sequencing.

### 2.3. Mammalian Cell Culture

The cell culture reagents were purchased from Lonza (Verviers, Belgium). The human BC cell SUM159PT was obtained by the American Tissue Culture Collection (ATCC, Rockville, MD, USA). Cells were routinely grown in monolayer culture and maintained at 37 °C in an atmosphere of 5% CO_2_. Chinese Hamster Ovary (CHO) cells were cultivated in Dulbecco’s Modified Eagle’s Medium (DMEM)/Ham’s F12 1:1 (PAN-Biotech, Aidenbach, Germany) that was supplemented with 5% fetal calf serum (FCS) (Sigma-Aldrich, Taufkirchen, Germany) and then maintained at 37 °C and 5% CO_2_. The SUM159PT cells were grown in DMEM/F12 (1:1) containing 5% heat-inactivated fetal calf serum (FCS), 2 mM L-glutamine, 1 μg/mL hydrocortisone, and 5 μg/mL insulin.

### 2.4. Transfection of CHO Cells and SUM159PT Cells

#### 2.4.1. Transfection of CHO Cells

For transient transfections, the CHO cells were cultivated in 10 cm dishes until they reached 70–80% confluency. A mixture of 12 µL PEI MAX (Polysciences, Warrington, PA, USA) and 12 µg of plasmid DNA was prepared in 1.2 mL Opti-MEM (Gibco; Thermo Fisher Scientific, Waltham, MA, USA), incubated for 20 min. at room temperature and then added drop-wise to a cell culture containing 12 mL of culture medium. After 6 h, the transfections were stopped by removal of the transfection mixture and the addition of fresh culture medium. Transfections in 24-well plates were performed accordingly while using a mixture of 0.5 µL PEI and 0.5 µg DNA in 50 µL of OptiMEM that was added to cells that were maintained in 500 µL of culture medium.

#### 2.4.2. Transfection of SUM159PT cells

- siRNA transfection

The depletion of CASD1 was performed using siRNA strategy by a double transfection. The second transfection was performed 48h after the first one using the same conditions. Cells were grown in six-well plates and transfections were performed with 2 μM of siRNA-targeting CASD1 (L-016926-01-0010, Horizon, Cambridge, UK) or a scramble sequence and 8 μL RNAimax (#137781, Thermo-Fisher Scientific, Waltham, Massachusetts, USA) in 1 mL of UltraMem (Lonza, Basel, Switzerland). After 5 h, transfection was stopped by adding 1 mL of DMEM/F12 media that was supplemented with 5% FCS. The cells were collected at 72 h for quantitative polymerase chain reaction (qPCR) and immunocytochemistry experiments.

- Transfection of SUM159PT cells with CASD1 or GD3 synthase-encoding expression vector

The transfection of SUM159PT cells was performed with RNAimax transfection reagent (#137781, Thermo-Fisher Scientific). Cells were grown in six-well plates, washed twice with UltraMem, and then transfected with 2 μg of plasmid DNA and 4 μL of RNAimax in 1 mL of UltraMem (Lonza). After 5 h, transfection was stopped by adding 1 mL of DMEM/F12 media that was supplemented with 5% of FCS. For the selection of stable transfectants, 500 μg/mL of hygromycin was added per well 48 h post-transfection. The clones were isolated by limited dilution. Positive clones were selected by qPCR and immunocytochemistry-confocal microscopy experiments.

### 2.5. CRISPR/Cas-Mediated Genome Editing

The CHO cells carrying a selective *Casd1* gene knockout (CHOΔ*Casd1*) were generated by introducing a frameshift mutation in exon 2 of *Casd1* by CRISPR/Cas9-mediated genome editing. Exon 2 of hamster *Casd1* corresponds to exon 3 of human *CASD1* and encodes the active site serine. A plasmid encoding a respective Casd1-specific guide RNA was generated on the basis of the bicistronic vector pX330-U6-Chimeric_BB-CBh-hSpCas9, which was a gift from Feng Zhang (Addgene plasmid # 42230; http://n2t.net/addgene:42230 (accessed on 1 April 2021); RRID:Addgene_42230). Following the protocol that was provided in Cong et al. ([27], the exon 2-specific target sequence 5′-TTGCATTTATCGGAGATTCCAGG-3′ (PAM sequence underlined) was inserted into the BbsI sites of the vector. The final plasmid allowed the co-expression of the RNA-guided nuclease Cas9 from Streptococcus pyogenes and the Casd1-specific guide RNA. The transient transfections in CHO cells were performed in 24-well plates using 0.375 µg of the CRISPR/Cas9-plasmid and 0.125 µg of a reporter plasmid (pEGFP-C1, Clontech, San Jose, CA, USA) that allowed for the expression of the enhanced green fluorescent protein (EGFP). After 24 h, the cells were cloned by limiting dilution and colonies grown from EGFP-expressing single-cell clones were expanded and screened for frameshift mutations. This included the amplification of the target region by PCR using two primer sets (5′-GCTGTGCCTAACAGTTTG-3′/5′-TGGCAAGTTTTTCCATGAG-3′ and 5′-TGAAGCAAAGAATTGCCTTGTAGA-3′/5′-CTTATTTCCTTCTTCTTTAAACTGGG-3′) and sequencing of the obtained PCR product. CHO clones carrying homozygous or heterozygous frameshift mutations in exon 2 of *Casd1* were subcloned by limiting the dilution and then re-analyzed. In this step, frameshift mutations were confirmed on the genomic level, as described above, and additionally verified on the transcript level by amplification of Casd1 transcripts by RT-PCR and analysis of the PCR products by sequencing. As gene-specific primers, the following multiple intron-spanning primer pair was used: 5′-ATGTTCACAACGCCACGG-3′ (exon 1) and 5′-CAGGAACCATCCACAGGC-3′ (exon 8). The CHOΔ*Casd1* clone used in this study contains a 2 bp insertion on one allele and a 4 bp deletion on the second allele. Both of the frameshift mutations occurred at the 5′-end of the triplet encoding Asp-60. This eliminated the triplet that encodes the catalytic residue Ser-61 and it resulted in the formation of a premature stop codon in exon 2 (see Supplementary Figure S1).

### 2.6. Production of the Sialyl-9-O-Acetyltransferase NeuD of Campylobacter Jejuni

The coding sequence of NeuD (orf11) was amplified from the genomic DNA of the *Campylobacter jejuni* (*C. jejuni*) strain MK104 (ATCC 43446) in a PCR reaction with the primers 5′-CGCCGCGGATCCGAAAAAATAACCTTAAAATGC-3′ and 5′-GTCCGCTCGAGTTAAAATAGATTAAAAATTTTTTTTGATTTTAG-3′. The obtained PCR product was ligated into the BamHI and XhoI sites of a pET32a (Novagen) vector that carries a sequence encoding the maltose binding protein (MBP), an (S)3(N)10-linker, and a thrombin cleavage site (LVPRGS) that was inserted into the NdeI and XhoI sites, with the last two triplets encoding the most C-terminal amino acids of the cleavage site (GS) creating a unique BamHI restriction site. The identity of the resulting construct was confirmed by sequencing and the encoded MBP-NeuD fusion protein was expressed in *E. coli* BL21(DE3). The transformed cells were cultivated at 37 °C in Power Broth (AthenaES) until an optical density at 600 nm of 1.5 was reached. The expression was induced with 1mM isopropyl-β-D-thiogalactopyranoside (IPTG) and cultivation at 15 °C for 20 h. The cells were harvested and resuspended in binding buffer (20 mM Tris-HCl pH 7.4, 200 mM NaCl, 1 mM EDTA) containing 40 µg/mL bestatin, 1 µg/mL pepstatin, and 1 mM PMSF, and they were disrupted by sonication. The recombinant protein was purified on 1 mL MBPTrap HP columns (GE Healthcare, Boston, MA, USA) using 10 mM D-(+)-maltose in binding buffer for elution. Affinity purified protein was dialyzed against 50 mM MES pH 7.0 containing 100 mM NaCl (Slide-A-Lyzer, ThermoFisher, 3.5 kDa cutoff) and concentrated using an Amicon Ultra-4 centrifugal filter device (Merck Millipore, Darmstadt, Germany; 50 kDa cutoff).

### 2.7. Generation of 9-O-Acetylated Gangliosides as Standards for TLC

The 9-*O*-acetylated forms of GD2 and GD3 were generated by enzymatic in vitro synthesis using NeuD from C. jejuni, which allows the site-selective introduction of an *O*-acetyl group at position C9 of a terminal α2,8-linked sialic acid [28]. The *O*-acetylation of GD3 (Sigma-Aldrich, 345752) and GD2 (Sigma-Aldrich, 345743) was performed according to Romero-Ramirez and co-workers [29]. The reaction was stopped by adding an equal volume of methanol and the gangliosides were purified on Chromabond C18 columns (Macherey-Nagel, Düren, Germany), dried under a nitrogen stream, and then dissolved in chloroform/methanol (1:2, *v*/*v*).

### 2.8. Extraction of Gangliosides

The total gangliosides were extracted from transfected CHO cells by mixing 10^7^ cells with 3 mL chloroform/methanol (1:2, *v*/*v*) and sonic dispersion. After twenty pulses being given by a Sonifier S-450 equipped with a cup horn (Branson), the samples were incubated for 15 min. in a bath sonicator. Debris was removed by centrifugation (1600× *g* for 10 min.) and the supernatant was transferred into a new tube. After adjusting a final ratio of chloroform/methanol/water of 4:8:5 (*v*/*v*/*v*), the samples were centrifuged (1600× *g* for 10 min.) and the upper phase containing the ganglioside fraction was desalted on a Chromabond C18 column (Macherey-Nagel, Düren, Germany). The gangliosides were dried under a nitrogen stream, dissolved in 20 µL of chloroform/methanol (1:2, *v*/*v*) and stored at −20 °C.

### 2.9. High-Performance Thin-Layer Chromatography (HPTLC) and Immunostaining

The total gangliosides of an equivalent of 2 × 10^6^ cells or 0.2 µg of the indicated ganglioside standards were spotted on Nano-DURASIL-20 (0.2 mm silica gel 60) HPTLC plates (Macherey–Nagel) and then chromatographed in chloroform/methanol/H_2_O (50:40:10, *v*/*v*/*v*) containing 0.05% calcium chloride. HPTLC plates were dried and chromatographed twice in 0.5% poly(isobutyl methacrylate) (Sigma-Aldrich, Taufkirchen, Germany) in hexane, which was prepared from a 25% stock solution in chloroform (*w*/*v*). The plates were dried and incubated overnight at 37 °C in PBS. After blocking with 2% BSA (*w*/*v*) in PBS for 1 h at room temperature, the plates were incubated with the following primary antibodies diluted in PBS: mouse IgG3 anti-9-*O*AcGD2 mAb 8B6 (10 µg/mL; OGD2 Pharma), mouse IgM anti-9-*O*AcGD3 mAb M-T6004 (1:40; Thermo Scientific, MA1-34707), mouse IgG2a anti-GD2 mAb ME361 (15 µg/mL; Kerafast, Boston, MA, USA; EWI023), or mouse IgG3 anti-GD3 mAb R24 (10 µg/mL; purified by protein A affinity chromatography from cell culture supernatant of R24 hybridoma cells ATCC HB-8445). HPTLC plates were washed three times with PBS and then incubated for 1 h at room temperature with goat anti-mouse IgM IRDye 800CW-conjugate (1:20,000; 926-32280; LI-COR Biosciences, Lincoln, NE, USA) or goat anti-mouse IgG IRDye 800CW-conjugate (1:10,000; 926-32210; LI-COR Biosciences, Lincoln, NE, USA). The HPTLC plates were washed with PBS and bound antibodies were detected by infrared imaging using an Odyssey Imaging System (LI-COR Biosciences).

### 2.10. RNA Extraction, cDNA Synthesis and qPCR

Gene expression was evaluated using real-time qPCR analysis after RNA extraction and cDNA synthesis. The total RNA was extracted from cell lines using the Nucleospin RNA II kit (Macherey-Nagel, Düren, Germany). The amount of extracted RNA was quantified using a DeNovix DS-11 spectrophotometer (DeNovix Inc., Wilmington, DE, USA) and the purity of the RNA was checked by the ratio of the absorbance at 260 and at 280 nm. The total RNA was subjected to reverse transcription using the Maxima First Strand cDNA Synthesis Kit (ThermoFisher Scientific, Villeneuve d’Ascq, France). The oligonucleotide sequences (Eurogentec, Seraing, Belgium) used as primers for the PCR reactions are described in [17]. qPCR and subsequent data analysis were performed using the Mx3005p Quantitative System (Stratagene, La Jolla, CA, USA). PCR reaction (25 µL) contained 12.5 µL of the 2X Brilliant SYBR Green qPCR Mastermix (Thermo Fischer Scientific, Rockford, IL, USA), 300 nM of primers, and 4 µL of cDNA (1:40). DNA amplification was performed using the following thermal cycling profile: initial denaturation at 94 °C for 10 min., followed by 40 cycles of amplification (denaturation at 94 °C for 30 s, annealing at Tm for 30 s, and extension at 72 °C for 30 s), and a final extension at 72 °C for 5 min. Hypoxanthine-guanine PhosphoRibosylTransferase (HPRT) gene was selected in former experiments as the best gene to normalize the expression of our genes of interest [17]. The fluorescence monitoring occurred at the end of each cycle. The analysis of amplification was performed using the Mx3005p software (version 3005p). For each primer pair, the specificity of the amplification was checked by recording the dissociation curves. The efficiency of amplification was checked by serial dilutions of cDNA from SK-MEL-28 cells, and it was between 97 and 102%. All of the experiments were performed in triplicate and the quantification of mRNA relative expression was performed as described in [17].

### 2.11. Immunocytochemistry and Confocal Microscopy

The transfected cells were grown on glass coverslips fixed for 15 min in 4% paraformaldehyde in 0.1 M sodium phosphate buffer. Cells were washed thrice with PBS and membrane permeabilization was performed in 5 μg/mL digitonin in PBS for 20 min. After saturation in blocking buffer, the cells were incubated with either with the anti-GD2 or anti-OAcGD2, or anti-V5- tag mAbs at 20 μg/mL for 1h followed by the secondary antibody for 1h. Cells were washed and then mounted in fluorescent mounting medium (Dako, Carpetaria, CA, USA). The stained slides were analyzed under a Zeiss LSM 700 confocal microscope. The same settings were used for all acquisitions to ensure the comparability of the data obtained.

### 2.12. MTS Assay

Cell growth was analyzed using the MTS reagent (Promega, Charbonnières-les-bains, France) according to the manufacturer’s instructions. Briefly, the cells were seeded in 96 well plates in 0%, 1% or 5% FCS containing media in which MTS reagents were added. The proliferation rate was measured by the absorbance of MTS reagent at 490 nm at 24 h, 48 h, 72 h, and 96 h after seeding.

### 2.13. Transwell Assays

The migration and invasion properties of cells were measured by transwell assays using migration chambers or invasion chambers (Dutscher, Brumath, France). Cells were seeded in 24-well plates containing either migration or invasion chambers in serum-free media. After 24 h incubation at 37 °C, the cells were fixed 4% paraformaldehyde in 0.1 M sodium phosphate buffer and non-migratory/invasive cells were swapped with cotton swabs. The nuclei were counterstained with DAPI and membrane were mounted on the slide with fluorescent mounting medium (Dako, Carpetaria, CA, USA). Nuclei were counted under Leica microscope.

### 2.14. Statistical Analysis

The statistical difference was assessed using unpaired *t*-test or ordinary one-way ANOVA. The unpaired *t*-test was used to compare the differences between means with the standard error difference computed by the combining the standard errors of two groups. One-way ANOVA was used for comparing the differences among group means with the pooled standard deviations of the groups that are defined by one factor.

## 3. Results

### 3.1. CASD1 Is Essential for the 9-O-Acetylation of GD2

**Figure 3:**
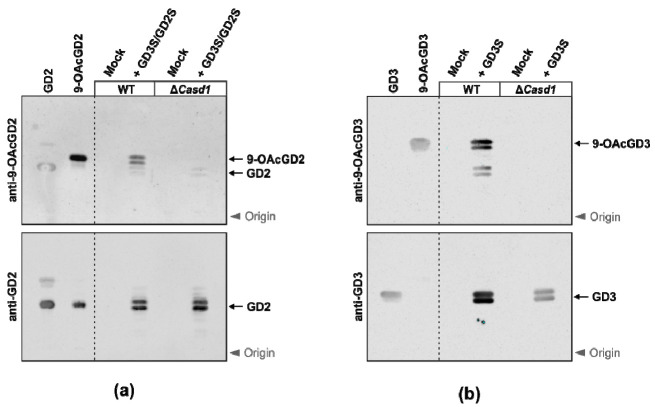
CASD1 induces the *O*-acetylation of GD2 and GD3 in CHO cells. Total gangliosides were extracted from CHO-WT or CHOΔ*Casd1* cells transfected with empty vector (mock) or a plasmid encoding the indicated synthases. The gangliosides were separated by thin-layer chromatography and stained with the indicated antibodies. Pure gangliosides and their in vitro generated 9-*O*-acetylated forms were used as standards (left panel). Please note that the *O*AcGD2 standard contains residual amounts of GD2. (**a**) CASD1-dependent formation of 9-*O*AcGD2. (**b**) CASD1-dependent formation of 9-*O*AcGD3.

Prior to deciphering the role of CASD1 in BC cells, we dissected the biosynthesis of 9-*O*AcGD2 in CHO cells, a well-defined cellular system. CHO cells mainly display the mono-sialyl ganglioside GM3, are easy to transfect, and are known to produce 9-*O*AcGD3 upon the expression of GD3S [30]. Using CRISPR/Cas9-mediated genome editing, we generated CHOΔ*Casd1* cells by introducing a frameshift mutation in exon 2 (Supplementary Figure S1). To produce GD2, CHO wild type (WT) and CHOΔ*Casd1* cells were transiently transfected with a bicistronic plasmid that allows the co-expression of GD3S and GD2S. The total gangliosides were extracted from transfected cells and analyzed by thin-layer chromatography (TLC). Upon transfection of the expression plasmid, but not of empty vector (mock), GD2 was detected in both CHO-WT and CHOΔ*Casd1* cells (Figure 3a, lower panel). The formation of 9-*O*AcGD2 was observed in CHO-WT, but not in *Casd1*-deficient cells (Figure 3a, upper panel), demonstrating that the biosynthesis of 9-*O*AcGD2 critically relies on CASD1. In addition, we monitored the formation of GD3 and 9-*O*AcGD3 in GD3S expressing CHO cells (Figure 3b). In line with the previous data obtained in HAP-1 cells [25], the deletion of *Casd1* in CHO cells prevented the formation of 9-*O*AcGD3.

### 3.2. Transient Modulation of CASD1 Expression in SUM159PT BC Cells

#### 3.2.1. CASD1 Expression Is Ubiquitous among Breast Cancer Cells

**Figure 4:**
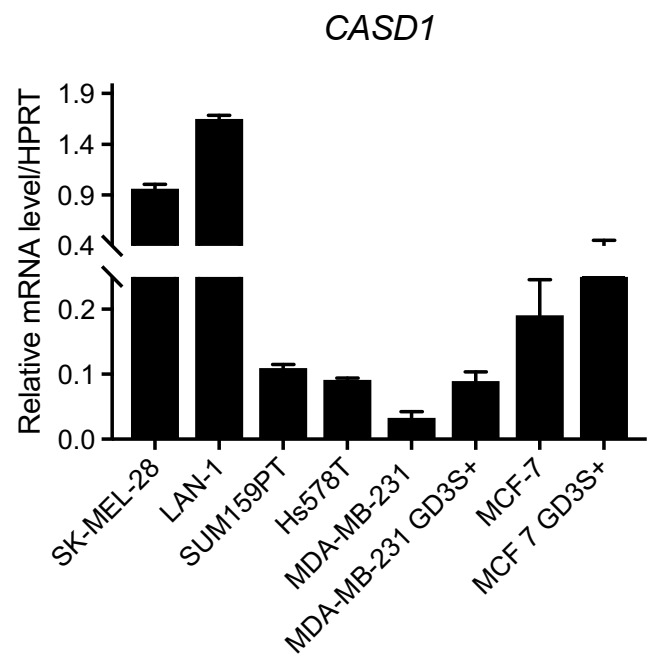
*CASD1* expression in neuro-ectoderm derived cancer cells. *CASD1* mRNA expression was determined by qPCR in BC cell lines. SK-MEL-28 melanoma cell line and LAN-1 neuroblastoma cell line were used as controls. The results were normalized to the expression of *HPRT* (hypoxanthine phosphoribosyl transferase) mRNA. Each bar represents the mean ± SD of *n* = 3 experiments.

Our results in CHO cells suggest that the expression of 9-OAcGD2, which is the major *O*-acetylated ganglioside species in BC cells, is CASD1 dependent. We next studied *CASD1* expression in breast cancer cells. The human protein atlas reveals that *CASD1* is expressed in almost all healthy and cancer tissues (http://www.proteinatlas.org/ENSG00000127995-CASD1/tissue, accessed on 1 April 2021). We performed qPCR experiments to quantify the expression of *CASD1* in different BC cells. We used SUM159PT, Hs578T, and two clones derived from MDA-MB-231 (MDA-MB-231 GD3S+) and MCF-7 (MCF-7 GD3S+) BC cell lines overexpressing GD3 synthase and high levels of complex gangliosides [17]. SK-MEL-28 melanoma cells and LAN-1 neuroblastoma cells expressing high levels of *O*-acetylated gangliosides were used as the controls. Our results presented in Figure 4 indicate that *CASD1* expression is ubiquitous among BC cells confirming the human protein atlas data. Among the BC cell lines tested, *CASD1* is more expressed in MCF-7 and MCF-7 GD3S+, as compared to MDA-MB-231, MDA-MB-231 GD3S+ SUM159PT, and Hs578T cells. The level of *CASD1* expression is distinctly higher in SK-MEL-28 and LAN-1 compares to BC cells. SUM129PT, a triple negative BC cell line that is derived from anaplastic carcinoma, was chosen for this study [28]. Our previous data show a moderate expression of GD2 and *O*AcGD2 [17] and CASD1 (Figure 4), suggesting that SUM159PT is suitable for both the depletion and overexpression of CASD1.

#### 3.2.2. Reduction of CASD1 Expression

**Figure 5:**
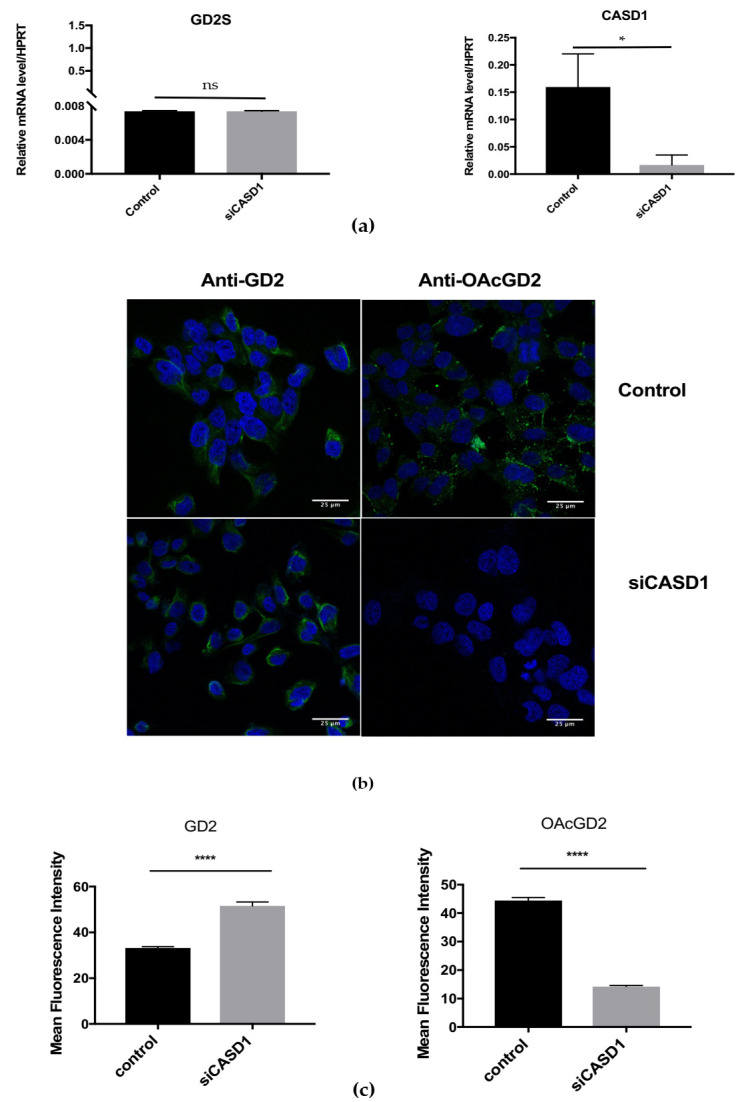
Reduced *O*AcGD2 expression in SUM159PT cells depleted for *CASD1* expression using siRNA strategy. (**a**) qPCR quantification of GD2S and *CASD1* expression in transiently transfected and control SUM159PT cells (*n* = 3). Results were normalized to the expression of *HPRT* mRNA. (**b**) Representative images of the analysis of GD2 and *O*AcGD2 expression in SUM159PT cells using immunocytochemistry and confocal microscopy (*n* = 3). Cells were incubated with an anti-GD2 and the anti-*O*AcGD2 mAb and gangliosides were visualized using IgG conjugate Alexa Fluor 488. The nuclei were counterstained with DAPI. All of the images were taken in the same settings. Scale bar: 25 µm. (**c**) Quantification of the mean fluorescence intensity of GD2 and *O*AcGD2. Statistical difference using unpaired *t*-test: * *p* < 0.5; **** *p* < 0.0001; ns: not significant.

The reduction of *CASD1* expression in SUM159PT was performed by transient transfection using siRNA strategy. The expression levels of GD2S (*B4GALNT1*) and *CASD1* genes were determined by qPCR experiments and then normalized to *HPRT* gene expression. The transfected cells exhibit a decrease of *CASD1* gene expression (up to 90%), while GD2 synthase gene expression is unchanged when compared to control cells (Figure 5a). The effect of CASD1 depletion on *O*AcGD2 expression was evaluated by immunofluorescence and confocal microscopy experiments. *O*AcGD2 expression is reduced in *CASD1*-depleted cells as compared to control cells (Figure 5b). The mean fluorescence intensity calculated based on multiple images shows that transfected cells exhibit a 75% decrease in *O*AcGD2 expression compared to control cells (Figure 5c). We conclude that a 50% reduction of *CASD1* gene expression leads to a 75% decrease of *O*AcGD2 expression in the transiently transfected cells when compared to SUM159PT control cells. The stable depletion of *CASD1* expression using shRNA strategy has been performed twice. Nevertheless, the transfected cells did not grow after several passages in antibiotic-containing medium (data not shown), and stable *CASD1* depletion could not be achieved in SUM159PT BC cells.

#### 3.2.3. Transient Overexpression of CASD1 in SUM159PT BC Cells

**Figure 6:**
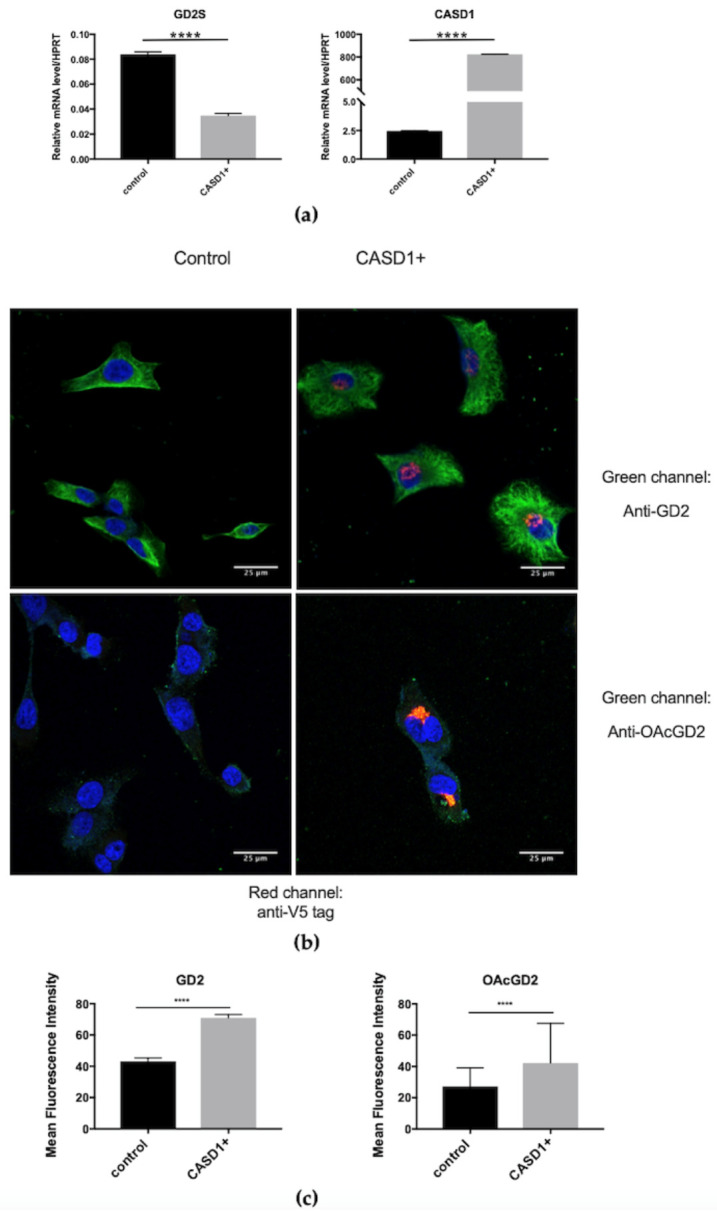
Increased *O*AcGD2 expression in *CASD1* overexpressing SUM159PT cells using plasmid transfection (CADS1+). (**a**) qPCR quantification of GD2S and *CASD1* genes in transiently transfected SUM159PT cells (*n* = 3). Results were normalized to the expression of *HPRT* mRNA. (**b**) Representative images of the analysis of GD2, *O*AcGD2 and V5-tag expression in BC cells by immunochemistry and confocal microscopy (*n* = 3). The cells were incubated with anti-V5-tag and either anti-GD2 or anti-*O*AcGD2 mAbs. Gangliosides were visualized using IgG conjugated-Alexa Fluor 488 and V5-tag using IgG conjugated-Alexa Fluor 546. The nuclei were counterstained with DAPI. All of the images were taken in the same settings. Scale bar: 25 µM. (**c**) Quantification of mean fluorescence intensity of GD2 and *O*AcGD2. Statistical difference using unpaired *t*-test: **** *p* < 0.0001; ns: non-significant.

The overexpression of *CASD1* (CASD1+) in SUM159PT cells was performed using a plasmid that allows the expression of human CASD1 with an N-terminal V5-epitope. In these experiments, *CASD1* and GD2 synthase (GD2S) gene expression was assessed by qPCR experiments and the effect of *CASD1* overexpression on *O*AcGD2 expression was studied by immunocytochemistry and confocal microscopy. *CASD1* mRNA expression level shows approximately a 3000-fold increase in transfected cells when compared to the control cells. Interestingly, GD2 synthase expression decreases significantly between the controls and transfected cells (Figure 6a). Representative images from immunocytochemistry analysis show the efficiency of transfection using an anti-V5-tag antibody (red channel) and ganglioside expression with either anti-GD2 or anti-*O*AcGD2 antibodies (green channel). *CASD1* transfected cells exhibit an increase in *O*AcGD2 and GD2 expression when compared to control cells (Figure 6b). The mean fluorescence intensity quantified for each condition shows that the overexpression of *CASD1* increases both GD2 and *O*AcGD2 expression by 60% and 55%, respectively (Figure 6c). We conclude that, as observed for the transient inhibition of *CASD1* gene expression, the transient overexpression of *CASD1* in SUM159PT shows an effect on *O*AcGD2 expression. Stable overexpression was considered because the stable depletion of CASD1 by shRNA in SUM159PT cells remained unsuccessful (data not shown).

### 3.3. Biological Properties of SUM159PT CASD1+

#### 3.3.1. Stable Overexpression of CASD1 in SUM159PT BC Cells

**Figure 7:**
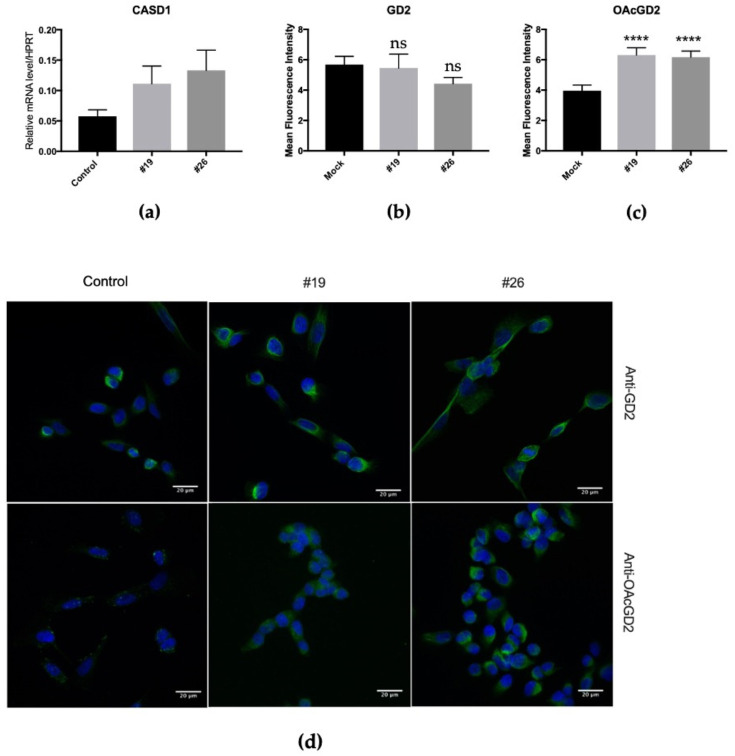
The expression of *CASD1* mRNA and quantification of *O*AcGD2 and GD2 expression in SUM159PT CASD1+ clones. (**a**) RT-qPCR quantification of *CASD1* gene expression in stably transfected and control SUM159PT cells (*n* = 3). Results were normalized to *HPRT* mRNA expression. (**b**) Quantification of mean fluorescence intensity of GD2. Statistical difference using unpaired *t*-test: **** *p* < 0.0001. (**c**) Quantification of mean fluorescence intensity of *O*AcGD2. Statistical difference using unpaired *t*-test: **** *p* < 0.0001. (**d**) Representative images of the analysis of GD2 and *O*AcGD2 in selected clones (#19, #26) as compared to control by immunocytochemistry and confocal microscopy (*n* = 3). Cells were incubated with anti-GD2 or anti-*O*AcGD2 mAbs. Gangliosides were visualized using IgG conjugated-Alexa Fluor 488. The nuclei were counterstained with DAPI. All images were taken in the same settings. Scale bar: 25 µM.

Stable transfectants overexpressing *CASD1* (SUM159PT CASD1+) have been produced using the plasmid pcDNA3.1 V5-tag-CASD1-cMyc and clones were isolated after antibiotic selection and limiting dilution cloning. Twelve clones were maintained during proliferation monitoring from the 28 clones pre-selected. *CASD1* expression levels in these clones were assessed by qPCR experiments, confirming the overexpression of *CASD1* in CASD1+ clones compared to controls (data not shown). The selection of CASD1+ clones among the 12 isolated clones has been performed by the analysis of GD2 and *O*AcGD2 expression using immunocytochemistry and confocal microscopy. Two CASD1+ clones exhibiting high *CASD1* gene expression and *O*AcGD2 ganglioside expression were used to study the biological properties. Figure 7 depicts the level of expression of *CASD1*, *O*AcGD2, and GD2 of the two selected clones (clone #19 and clone #26). *CASD1* mRNA expression was two-fold and three-fold increased in clone #19 and in clone #26 as compared to control cells, respectively (Figure 7a). The quantification of mean fluorescence intensity for GD2 and *O*AcGD2 staining in the different clones shows that the expression of GD2 remained unchanged (Figure 7b,d) whereas *O*AcGD2 expression increased in clones #19 and #26 as compared to control cells (Figure 7c,d).

#### 3.3.2. Biological Properties of SUM159PT CASD1+

**Figure 8:**
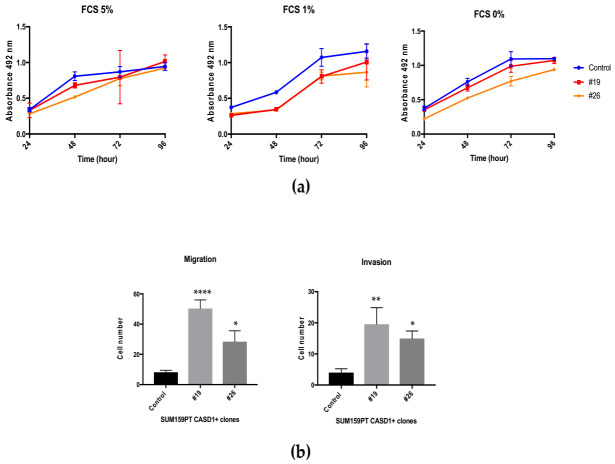
Biological properties of SUM159PT CASD1+ clones. (**a**) The growth of control and SUM159PT CASD1+ #19 and #26 clones was assessed after 0 h, 24 h, 48 h, 72 h, and 96 h using MTS reagent (Promega, Madison, WI, USA) in media containing 5%, 1% or 0% of fetal calf serum (FCS). (**b**) The migration and invasion capabilities of control and SUM159PT CASD1+ clones #19 and #26 were assessed after 48 h by Transwell assay in serum free media. Statistical difference using one-way Anova: **** *p* < 0.0001; ** *p* < 0.002; * *p* < 0.02.

Biological properties of the SUM159PT CASD1+ clones were studied by MTS and Transwell assays, in order to assess their proliferation and migration/invasion capabilities, respectively. SUM159PT CASD1+ clones did not exhibit differential growth properties when compared to their control counterpart, regardless of the percentage of fetal calf serum in the culture medium (Figure 8a). However, both of the clones showed increased migration and invasion capabilities in serum free media (Figure 8b). The migration capabilities of SUM159PT CASD1+ clones increased up to five-fold in clone #19, while migration was increased up to two-fold in clone #26 as compared to control cells. The invasion activity of clone #26 and clone #19 was increased five- and three-fold compared to control cells, respectively (Figure 8b). However, CASD1+ clones maintained in culture lost the expression of GD2 and *O*AcGD2 two months after selection and did not allow further experiments (Supplementary Figure S2a,b).

#### 3.3.3. Effect of GD2S, GD3S, and CASD1 Expression on the Survival of BC Patients

**Figure 9:**
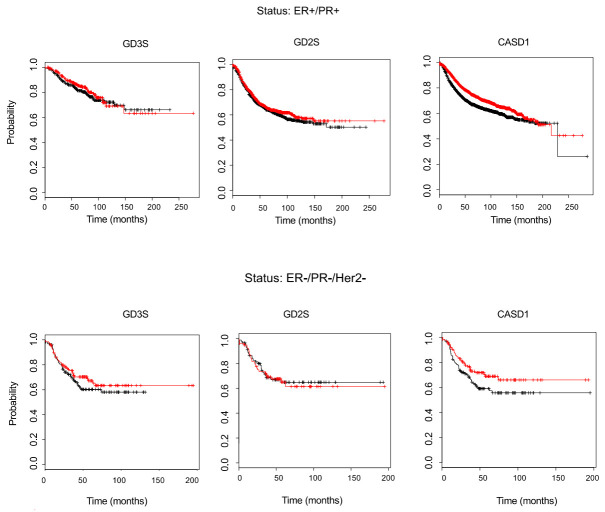
Kaplan–Meier survival plot in BC patients. The database integrates gene expression and clinical data simultaneously. The two patient cohorts are compared by a Kaplan–Meier survival plot, and the hazard ratio with 95% confidence intervals and log rank *p* value are calculated [31]. GD3 synthase, GD2 synthase, and *CASD1* expression are assessed in ER+/PR+ BC patients (**upper**) and ER-/PR-/Her2- BC patients (**bottom**). The black line represents the overall survival of patient with a low gene expression. The red line represents the overall survival of BC patients with high gene expression.

The biosynthesis of *O*AcGD2 and *O*AcGD3 requires both the expression of the glycosyltransferases GD2S and GD3S as well as the expression of the *O*-acetyltransferase CASD1. The effect of *B4GALNT1* (encoding GD2S), *ST8SIA1* (encoding GD3S), and *CASD1* expression levels in BC patients could be assessed by the Kaplan–Meier plotter database that integrates both gene expression and clinical data and allows for evaluating the overall survival of patients according to the gene expression levels. While GD2S and GD3S expression levels do not affect BC patients’ overall survival, regardless of their ER/PR/Her2 status (expression of the estrogen receptor/progesterone receptor/epidermal growth factor receptor 2), *CASD1* high expression is associated with a better survival of BC patients having ER–/PR–/Her2– status (Figure 9). These results may appear surprising because *O*-acetylated gangliosides are considered to be markers and therapeutic targets of interest in other neuro-ectoderm derived cancers, such as neuroblastoma and glioblastoma, and CASD1 is essential for *O*AcGD3 and *O*AcGD2 biosynthesis in different cell lines. However, very little is known regarding the diversity, the expression patterns, and the biological roles of *O*-acetylated gangliosides in cancer cell biology. More studies have to be performed to decipher the fine structures of *O*-acetylated gangliosides in breast cancer tissues, as well as to elucidate their roles in breast cancer cell biology.

## 4. Discussion

Ganglioside *O*-acetylation results from the enzymatic action of a SOAT on a sialic acid residue. CASD1 is the only human SOAT known up to date, which is involved in the *O*-acetylation of GD3 ganglioside [25]. *O*AcGD3 is one of the major and most studied *O*-acetylated gangliosides, but recent studies have highlighted the importance of *O*AcGD2 as a marker and therapeutic target of interest in neuro-ectoderm derived cancers, including BC [6,11,12,17,32,33]. Therefore, deciphering GD2 *O*-acetylation mechanisms and the involvement of CASD1 in *O*AcGD2 biosynthesis in BC is of utmost importance.

### 4.1. CASD1 Is Involved in GD2 9-O-Acetylation in CHO Cells and in SUM159PT Cells

In this study, we first used CHO cell lines that do not naturally express b-series gangliosides, as a model to study CASD1 activity on gangliosides. Ganglioside expression can be modulated in these CHO cell lines, either by overexpressing the GD3S required for GD3 expression, or both GD3S and GD2S for more complex ganglioside biosynthesis. Consequently, the CHO WT and CHOΔ*Casd1* cell lines are suitable models for studying CASD1 SOAT activity on different gangliosides. The use of these cell lines allowed us to conclude that no *O*-acetylated ganglioside was detected in CHOΔ*Casd1* cells, highlighting the critical role of CASD1 in both GD3 and GD2 9-*O*-acetylation. These data also suggest that CASD1 is the unique SOAT that is involved in GD3 and GD2 9-*O*-acetylation in CHO cells.

Because there are no BC cellular models available with a knockout for *CASD1*, the modulation of *CASD1* expression was adopted as the strategy to assess the potential SOAT activity of CASD1 on GD2 *O*-acetylation in SUM159PT BC cell line. The transient overexpression or depletion of *CASD1* in SUM159PT cells modulated *O*AcGD2 expression: RNAi silencing of *CASD1* induced a 70% decrease of *O*AcGD2 expression (Figure 5c), whereas *CASD1* overexpression increased *O*AcGD2 expression (50% increase) (Figure 5c and Figure 6c). The GD2 levels were increased both when *CASD1* is overexpressed or when *CASD1* is depleted (Figure 5c and Figure 6c). Our previous structural analysis had allowed identifying 9-*O*Ac*GD2* as the major *O*-acetylated ganglioside species. Altogether, these data suggest that CASD1 is involved in GD2 9-*O*-acetylation in BC cells, as demonstrated in CHO cells.

### 4.2. Influence of CASD1 and OAcGD2 on BC Cell Properties

Thirty clones overexpressing *CASD1* have been isolated and assessed for *O*AcGD2 expression. Two clones were selected according to their level of *O*AcGD2/CASD1 overexpression. These clones exhibited higher migrative and invasive capacities with no modification of their proliferation rates, suggesting a role of *O*AcGD2 in BC migration and invasion. Although *O*-acetylated gangliosides, such as *O*AcGD3 and *O*AcGD2, are now considered as TACAs, there are very little data in the literature regarding their roles in cancer cell biology. *O*AcGD3 protects leukemic blasts, Jurkat cells, and glioblastoma cells from apoptosis [34,35]. Moreover, increased levels of 9-*O*-acetylated Neu5Ac corresponding notably to elevated 9-*O*AcGD3 were detected in acute lymphocytic leukemia (ALL) cells that developed resistance against vincristine or nilotinib, two drugs with different cytotoxic mechanisms. The treatment of ALL cells by a sialate acetyl esterase that cleaved the 9-*O*-acetyl residues from sialic acids made these cells more sensitive to both drugs [36]. SIAE overexpression in hamster melanoma cells induced a loss of *O*AcGD3, altered cell morphology, a slower growth rate, and lower melanogenesis activity when compared to controls [37]. Previous studies suggest a role of *O*AcGD2 in cancer cell properties, for example an anti-*O*AcGD2 mAb c.8B6 monoclonal antibody inhibited glioblastoma and neuroblastoma cell proliferation in vitro and in vivo [13]. Here, we describe higher migrative and invasive capacities of SUM159PT clones overexpressing *CASD1* and 9-*O*AcGD2, with no modification in their proliferation rates. Importantly, *CASD1* overexpression could modulate the expression of other *O*-acetylated gangliosides or sialylated glycosphingolipids (Globo-, Lacto-/Neolacto-series), which could also modify the biological properties of cancer cells. The cellular mechanisms that are involved in increased malignant properties of SUM159PT cells with high *CASD1*/*O*AcGD2 expression need to be studied.

### 4.3. Cellular Models and Strategies Used for the Study of CASD1/OAcGD2 Expression

The maintenance of SUM159PT-CASD1+ clones in culture for 10 passages induced the loss of both CASD1 and of *O*AcGD2 overexpression, which suggests that an inducible overexpression system would be more suitable for studying the function of CASD1 in SUM159PT cells in a controlled manner. The difficulties that we encountered to keep stable SUM159PT clones overexpressing *CASD1* or to generate clones stably depleted for *CASD1* by shRNA (data not shown) raise questions concerning the choice of BC cells that were used for the study and the methods employed to generate stable clones. The results obtained by transient transfections in SUM159PT cells were an incentive to use the same cell line to generate stably transfected clones. The moderate *O*AcGD2 expression rate in SUM159PT cells was adapted to induce both an increase and a decrease of *O*AcGD2 expression, avoiding the use of two different cell models to study the effect of *CASD1* modulation on *O*AcGD2 expression. Cultured cancer cell lines are usually extrapolated to in vivo human tumors, and their importance as models for drug testing and translational study has been recognized by many biomedical and pharmaceutical companies, but one should keep in mind that they bear more chromosomic aberrations (especially an increased chromosome copy number) and different metabolic features when compared to normal cells.

CASD1 is ubiquitously expressed in all tissues and cells according to the Human Protein Atlas. In agreement, all of the BC cell lines tested in this study express *CASD1* at variable levels. Mahajan and co-workers recently generated a mouse strain in which *Casd1* was deleted in the germline. So far, only the hematopoietic system had been analyzed. In this initial study, all of the hematopoietic cells of the knockout mice did not show any specific phenotype, but lost 9-*O*-acetylated sialic acid species; these data suggest that CASD1 could be, in mice, the only SOAT that is involved in sialic acid-9-*O*-acetylation in erythroid, myeloid, and CD4 T lineages [26]. Baumann and coworkers demonstrated the role of CASD1 in GD3 *O*-acetylation using *CASD1* knockout HAP-1 cells that were edited by CRISPR Cas-9 technology [25]. CHO and HAP-1 cells deleted for *CASD1* both did not show any disturbance of their proliferative and growth capacities [26]. The absence of growth of SUM159PT cells that we observed in cells stably depleted for *CASD1* can be interpreted as an ineffective shRNA strategy for the depletion, which could be replaced by developing gene editing technologies approaches, such as CRISPR-Cas9, or by the absolute requirement of CASD1 for SUM159PT cell growth.

### 4.4. Influence of GD3S, GD2S and CASD1 Expression on the Overall Survival of BC Patients

The effect of *B4GALNT1* (encoding GD2S), *ST8SIA1* (encoding GD3S), and *CASD1* expression levels in BC patients were assessed by the Kaplan–Meier plotter database that integrates both gene expression and clinical data, and allows evaluating the overall survival of patients according to gene expression levels. While GD2S and GD3S expression levels do not affect BC patients’ overall survival, regardless of their ER/PR/Her2 status, the high expression of *CASD1* is associated with a better survival of BC patients having ER^-^/PR^-^/Her2^-^ status. However, these data should not be over-interpreted and additional experiments are required if one wants to establish a link between ganglioside *O*-acetylation and the survival of BC patients. The synthesis of *O*-acetylated gangliosides is complex, tightly regulated, and depends on glycosyltransferase/glycosidase expression pattern and substrate availability, including the availability of acetyl-CoA.

It is admitted that GD3S expression promotes proliferation, invasion, migration, and colony formation of breast cancer cells [9]. *In silico* analysis revealed a higher GD3S expression in ER^−^ than ER^+^ breast cancers; GD3S was also highly expressed in triple negative breast cancers when compared to other types of breast cancers. The elevated GD3S expression in triple negative breast cancer (TNBC) cells and tissues was associated with hypomethylation of the *ST8SIA1* gene [38]. TNBC lack expression of the estrogen receptor (ER), progesterone receptor (PR), and human epidermal growth factor receptor 2 (HER2), and it is considered as the most aggressive type of breast cancers. Interestingly, GD2 is a marker of breast cancer stem cells, but its expression is linked to high GD3S and not high GD2S expression. GD3S is highly expressed in GD2+ as well as in CD44hiCD24lo breast cancer stem cells, and interference with GD3S expression reduced the cancer stem cell (CSC) population and CSC-associated properties [39], again showing the key role of GD3S in GD2 levels. We previously used a combinatorial approach (ganglioside quantification by MALDI-TOF, RT-qPCR data for glycosyltransferase/*CASD1* gene expression, and the use of the “Ganglio-sphingolipid metabolism” pathway from WikiPathways) to perceive the relationships between gangliosides expressed and glycosyltransferase (GTs) gene expression between two breast cancer cell lines. In both cases (Hs 578T vs. MDA-MB-231, and MDA-MB-231 GD3S+ vs. MDA-MB-231), *O*-acetylated gangliosides appear to be upregulated independently of *CASD1* expression variations between two cell lines, and in a substrate-dependent manner. *O*-acetylated ganglioside expression, especially *O*AcGD2, appears to be not only dependent on the expression level of enzymes involved in the biosynthesis of gangliosides, but also (and mostly) on substrate availability [40]. Consequently, the combined expression of more genes and the synthesized gangliosides should be studied in breast cancer tissues to have a more global view and identify relevant markers.

For now, CASD1 is only mentioned in nine publications in Pubmed (NCBI, accessed on 1 April 2021), showing the limited knowledge available regarding the physiological role of CASD1. The difficulties that are encountered for cloning and isolation of SOAT render the deciphering of *O*-acetylated ganglioside biosynthesis mechanisms complicated. Our data indicate a role of CASD1 in GD2 *O*-acetylation in BC cells and confirm a CASD1-dependent pathway for both 9-*O*AcGD2 and 9-*O*AcGD3 in SUM159PT BC cells and in CHO cells. In addition, we observed increased tumorigenic properties of BC cells over-expressing *CASD1* and *O*AcGD2. Further studies are required to determine whether CASD1 has a more general role in *O*AcGD2 biosynthesis in cancers with high *O*AcGD2 levels, such as neuroblastoma and glioblastoma. Deciphering the biosynthetic pathways as well as the structural diversity and the biological roles of *O*-acetylated gangliosides in neuro-ectoderm derived cancer cells and tissues should allow identifying new markers or therapeutic targets for cancer treatment.

## Data Availability

Not applicable.

## Supplementary Materials

The following are available online at https://www.mdpi.com/article/10.3390/cells10061468/s1, Figure S1. Schematic representation of the hamster *Casd1* gene locus showing the target site used for CRISPR/Cas9-mediated genome editing in exon 2 (underlined in black) and the corresponding PAM site (underlined in red). Figure S2: Representative images of GD2 and *O*AcGD2 expression in selected SUM159PT CASD1+ clones after 2 months. GD2 and *O*AcGD2 expression was visualized in selected SUM159PT CASD1+ clones and control cells by immunochemistry and confocal microscopy (*n* = 3). (a) Images taken right after the clonal selection. (b) Images taken after 2 months of clonal selection. Cells were incubated with anti-GD2 or anti-*O*AcGD2 mAbs. Gangliosides were visualized using IgG conjugated-Alexa Fluor 488. The nuclei were counterstained with DAPI. All images were taken in the same settings.

## Abbreviations

BC	Breast cancer
CASD1	Cas 1 domain containing 1
CSC	cancer stem cell
ER	estrogen receptor
GD2S	GD2 synthase (B4GALNT1)
GD3S	GD3 synthase (ST8SIA1)
GT	glycosyltransferase
HER2	human epidermal growth factor receptor 2
mAb	monoclonal antibody
Neu5Ac	N-acetylneuraminic acid
*O*AcGD2	*O*-acetylated-GD2
*O*AcGD3	*O*-acetylated-GD3
PR	progesterone receptor
SIAE	sialate *O*-acetyl esterase
SOAT	sialate *O*-acetyl transferase
SNGH	serine-glycine-asparagine-histidine
TACA	tumor-associated carbohydrate antigen
TLC	thin-layer chromatography
TNBC	triple negative breast cancer
