# Supplementary material for: Role of Sialyl-O-Acetyltransferase CASD1 on GD2 Ganglioside O-Acetylation in Breast Cancer Cells
Source: Cells. 2021 Jun 11;10(6):1468. doi: 10.3390/cells10061468 (PMC8230688; doi:10.3390/cells10061468)
Supplement: Supplementary file 1 [file cells-10-01468-s001.zip › cells-1199891-supplementary.pdf]

cells

MDPI

Supplementary Material

Role of Sialyl-*O*-Acetyltransferase CASD1 on GD2 Ganglioside *O*-Acetylation in Breast Cancer Cells

Sumeyye Cavdarli [1], Larissa Schröter [2,†], Malena Albers [2,†], Anna-Maria Baumann [2], Dorothée Vicogne [1], Jean-Marc Le Doussal [3], Martina Mühlenhoff [2], Philippe Delannoy [1], and Sophie Groux-Degroote [1,*]

[1] Univ Lille, CNRS, UMR 8576-UGSF- Unité de Glycosylation Structurale et Fonctionnelle; sumeyye.cavdarli@univ-lille.fr (S.C.); dorothee.vicogne@univ-lille.fr (D.V.); philippe.delannoy@univ-lille.fr (P.D.)
[2] Institute of Clinical Biochemistry, Hannover Medical School, 30623 Hannover, Germany; Schroeter.Larissa@mh-hannover.de (L.S.); Albers.Malena@mh-hannover.de (M.A.); anna-maria.junemann@gmx.de (A.-M.B.); Muehlenhoff.Martina@mh-hannover.de (M.M.)
[3] OGD2 Pharma, IRS2—Nantes BIOTECH, 44200 Nantes, France; ledoussal@ogd2pharma.com
* Correspondence: sophie.groux-degroote@univ-lille.fr
† These authors contributed equally to the work.

Citation: Cavdarli, S.; Schröter, L.; Albers, M.; Baumann, A.-M.; Vicogne, D.; Doussal, J.-M.L.; Mühlenhoff, M.; Delannoy, P.; Groux-Degroote, S. Role of Sialyl-O-Acetyltransferase CASD1 on GD2 Ganglioside O-Acetylation in Breast Cancer Cells. *Cells* **2021**, *10*, 1468. https://doi.org/10.3390/cells10061468

Academic Editor: Sebastian P. Galuska, Rüdiger Horstkorte

Received: 12 April 2021
Accepted: 7 May 2021
Published: 11 June 2021

Publisher's Note: MDPI stays neutral with regard to jurisdictional claims in published maps and institutional affiliations.

Copyright: © 2021 by the authors. Licensee MDPI, Basel, Switzerland. This article is an open access article distributed under the terms and conditions of the Creative Commons Attribution (CC BY) license (http://creativecommons.org/licenses/by/4.0/).

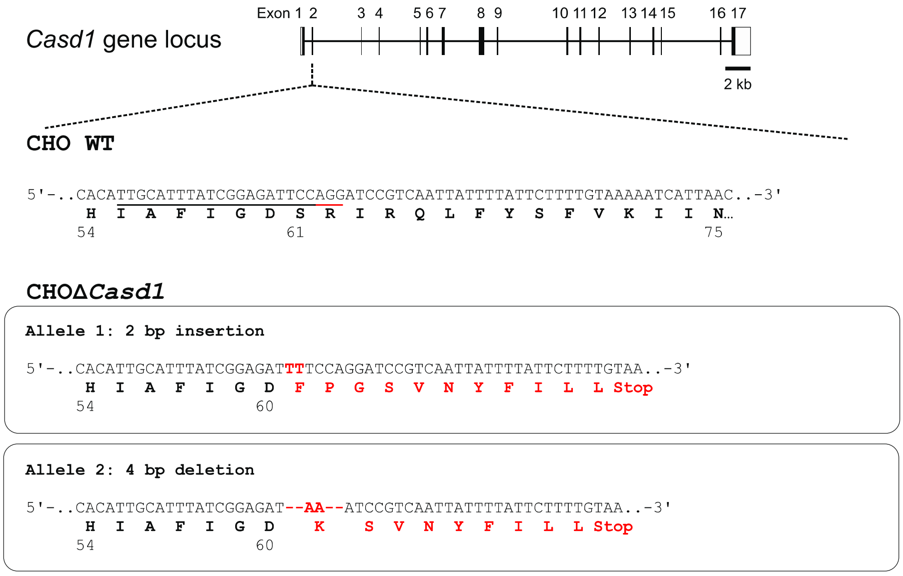

Figure S1. Schematic representation of the hamster *Casd1* gene locus showing the target site used for CRISPR/Cas9-mediated genome editing in exon 2 (underlined in black) and the corresponding PAM site (underlined in red). The CHOΔ*Casd1* clone used in this study harbors a 2 bp insertion on one allele and a 4 bp deletion on the other allele. Both frameshift mutations result in the loss of the catalytic residue Ser-61 and the formation of a premature stop codon.

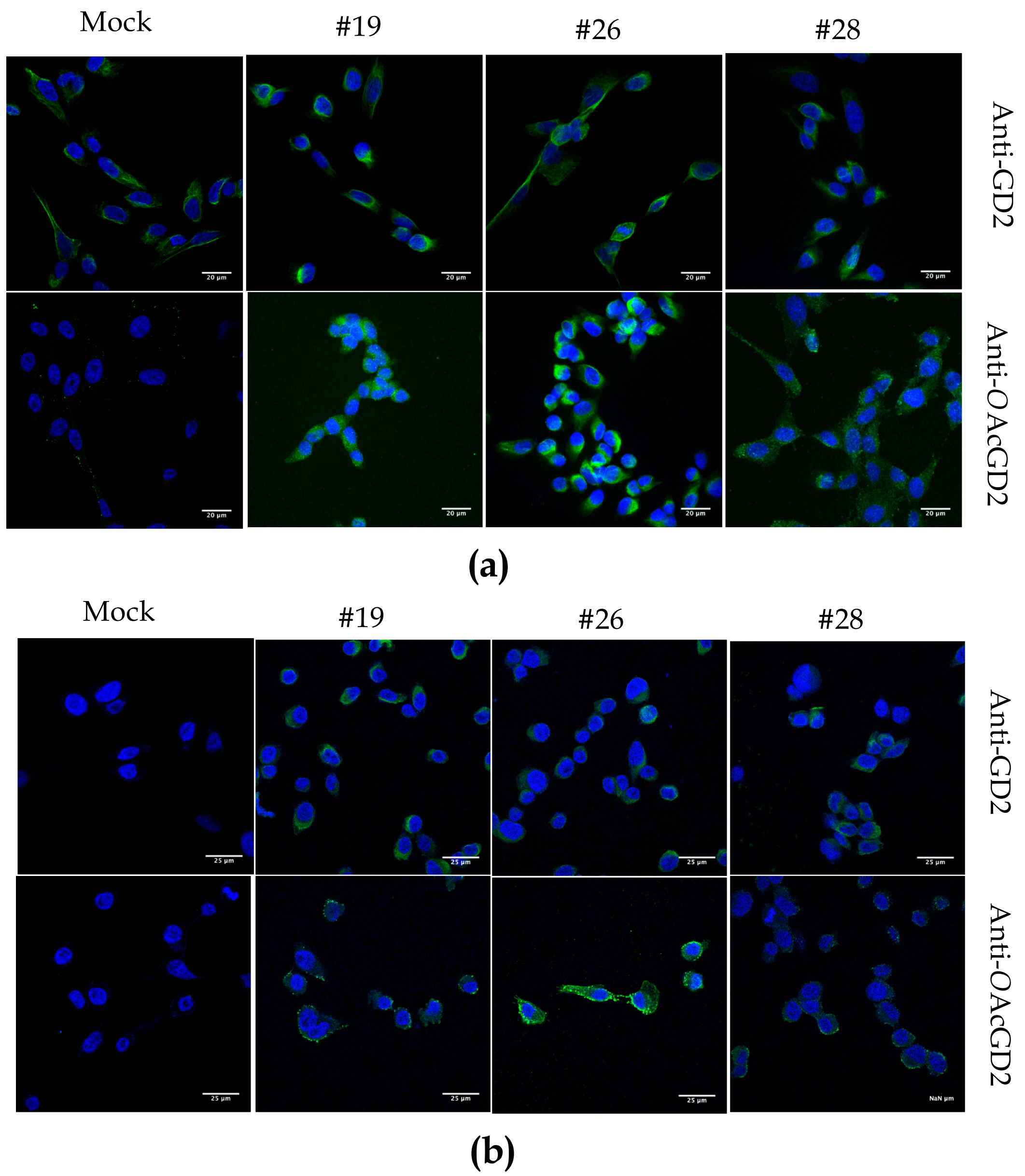

Figure S2. Representative images of GD2 and *O*AcGD2 expression in selected SUM159PT CASD1+ clones after 2 months. GD2 and *O*AcGD2 expression was visualized in selected SUM159PT CASD1+ clones and control cells by immunochemistry and confocal microscopy (n = 3). (a) Images taken right after the clonal selection. (b) Images taken after 2 months of clonal selection. Cells were incubated with anti-GD2 or anti-*O*AcGD2 mAbs. Gangliosides were visualized using IgG conjugated-Alexa Fluor 488. The nuclei were counterstained with DAPI. All images were taken in the same settings.
